# Correction: Modeling the dynamics of the COVID-19 population in Australia: A probabilistic analysis

**DOI:** 10.1371/journal.pone.0272762

**Published:** 2022-08-03

**Authors:** The PLOS ONE Staff

There is an error in affiliation 1 for authors Ali Eshragh, Peter Howley, and Elizabeth Stojanovski. The publisher apologizes for the error. The correct affiliation 1 is: School of Mathematical and Physical Sciences, University of Newcastle, Callaghan, NSW, Australia.
