# Supplementary material for: Modeling the dynamics of the COVID-19 population in Australia: A probabilistic analysis
Source: PLoS One. 2020 Oct 2;15(10):e0240153. doi: 10.1371/journal.pone.0240153 (PMC7531857; doi:10.1371/journal.pone.0240153)
Supplement: S1 Appendix — A brief overview of the underlying stochastic process used for modeling in this paper is presented in Appendix provided in Supplementary Materials. (PDF) [file pone.0240153.s001.pdf]

Modeling the dynamics of the COVID-19 population in Australia: A probabilistic analysis

Supplementary Materials

Ali Eshragh* Saed Alizamir† Peter Howley‡ Elizabeth Stojanovski‡

A Appendix: Background

In this appendix, we present a brief overview of the underlying stochastic process used for modeling in this paper. An extensive discussion can be found in [1, 2].

A.1 Partially-observable Continuous-time Markov Population Process

Suppose $\{X_t,\, t \geq 0\}$ is a continuous-time Markov population process with the unknown parameter vector $\boldsymbol{\theta}_t$. The vector $\boldsymbol{\theta}_t$ parameterizes the q-matrix (generator) $Q(\boldsymbol{\theta}_t)$ of the model. We restrict our attention to CTMPPs where the range of the random variable X_t includes nonnegative integers, and the initial value of this process, x_0, is known. Moreover, we suppose that the process is time-homogeneous, that is the conditional probability $\mathsf{P}_{(X_{t_2}|X_{t_1})}(x_{t_2}|x_{t_1})$ for any values of $t_2 > t_1 \geq 0$ depends only on x_{t_1}, x_{t_2} and $t_2 - t_1$.

In order to estimate the unknown parameter vector $\boldsymbol{\theta}_t$, we take n observations of $\{X_t,\, t \geq 0\}$ at times $0 < t_1 \leq \cdots \leq t_n$. Suppose that at each observation time t_i, we do not observe X_{t_i} directly, but rather only a random sample. This may be due to practical restrictions such as time or budget constraints which limit the ability to survey comprehensively, or might be because of an implicit component of the data collection process. A common model for the sampling is binomial, where the state of the system, or each component of the system, is observed with a probability p_t at observation time t. Definition 1 provides a formal definition of a *partially-observable continuous-time Markov population process.*

*School of Mathematical and Physical Sciences, University of Newcastle, NSW, Australia, and International Computer Science Institute, Berkeley, CA, USA. Email: ali.eshragh@newcastle.edu.au

†School of Management, Yale University, CT, USA.

‡School of Mathematical and Physical Sciences, University of Newcastle, NSW, Australia.

Definition 1 ([1]). *Consider the CTMPP $\{X_t,\, t \geq 0\}$ with the parameter vector $\boldsymbol{\theta}_t$. Suppose the random variables Y_t are defined such that the conditional random variable $(Y_t|X_t = x_t)$ follows the Binomial(x_t, p_t) distribution, that is*

$$\mathsf{P}_{(Y_t|X_t)}(y_t|x_t) = \binom{x_t}{y_t} p_t^{y_t}(1-p_t)^{x_t-y_t} \quad \text{for } y_t = 0, 1, \ldots, x_t\,.$$

Then the stochastic process $\{Y_t,\, t \geq 0\}$ is called a PO-CTMPP with the parameter vector $(\boldsymbol{\theta}_t, p_t)$.

Remark 1. It is readily seen that a PO-CTMP model with parameter vector $(\boldsymbol{\theta}_t, 1)$ reduces to a CTMP model with parameter vector $\boldsymbol{\theta}_t$.

In order to find the MLE of the unknown parameter vector $(\boldsymbol{\theta}_t, p_t)$, we first need to construct the likelihood function of partial observations, that is,

$$\mathsf{L}_{\boldsymbol{Y}_n}(\boldsymbol{y}_n; \boldsymbol{\theta}_t, \boldsymbol{p}_n) = \mathbf{Pr}(\boldsymbol{Y}_n = \boldsymbol{y}_n),$$

where the random vector $\boldsymbol{Y}_n := (Y_0, Y_{t_1}, \cdots, Y_{t_n})$, the realization vector $\boldsymbol{y}_n := (x_0, y_{t_1}, \ldots, y_{t_n})$, the probability vector $\boldsymbol{p}_n := (1, p_{t_1}, \ldots, p_{t_n})$, and $\mathbf{Pr}(Y_0 = x_0) = 1$.

Bean et al. [1] utilized the Conditional Bayes' Theorem [3] and derived the following analytical results:

Theorem 1 ([1]). *Consider a PO-CTMP process with the parameter vector $(\boldsymbol{\theta}_t, p_t)$.*

(i) The conditional p.m.f. of the true value of the underlying process given the partial observations is

$$\mathsf{P}_{(X_{t_n}|\boldsymbol{Y}_n)}(x_{t_n}|\boldsymbol{y}_n) = \frac{\varrho_n^{x_{t_n}}}{\sum\limits_{\ell=y_{t_n}}^{\infty} \varrho_n^{\ell}} \quad \text{for } x_{t_n} = y_{t_n}, y_{t_n}+1, \ldots,$$

where,

$$\varrho_n^{\ell} := ey_{t_n}! \binom{\ell}{y_{t_n}} p_{t_n}^{y_{t_n}}(1-p_{t_n})^{\ell-y_{t_n}} \sum_{j=y_{t_{n-1}}}^{\infty} \mathsf{P}_{(X_{t_n}|X_{t_{n-1}})}(\ell|j)\varrho_{n-1}^{j},$$

for $\ell = y_{t_n}, y_{t_n}+1, \ldots$, $n = 1, 2, \ldots$, and the initial conditions $\varrho_0^{x_0} = 1$ and $\varrho_0^{\ell} = 0$ for $\ell \neq x_0$.

(ii) The conditional p.m.f. $\mathsf{P}_{(Y_{t_{n+1}}|\boldsymbol{Y}_n)}(y_{t_{n+1}}|\boldsymbol{y}_n)$ *for* $y_{t_{n+1}} = 0, 1, 2, \ldots$ *equals*

$$\frac{\displaystyle\sum_{x_{t_{n+1}}=y_{t_{n+1}}}^{\infty} \sum_{x_{t_n}=y_{t_n}}^{\infty} \binom{x_{t_{n+1}}}{y_{t_{n+1}}} p_{t_{n+1}}^{y_{t_{n+1}}} (1-p_{t_{n+1}})^{x_{t_{n+1}}-y_{t_{n+1}}} \mathsf{P}_{(X_{t_n}|X_{t_{n-1}})}(x_{t_n}|x_{t_{n-1}})\, \varrho_n^{x_{t_n}}}{\displaystyle\sum_{\ell=y_{t_n}}^{\infty} \varrho_n^{\ell}},$$

for $n = 1, 2, \ldots$.

A.2 Partially-observable Pure Birth Process

A popular model in the class of CTMPP is the stochastic *pure birth process* (PBP). Let $\{X_t,\, t \geq 0\}$ be a time-homogeneous PBP, with the parameter λ_t (known as the birth/growth rate) at time t, and known initial population size of x_0. If $X_t = x_t$, then the *transition rate* equals $\lambda_t x_t$. It can be shown [4] that if the birth rate over a given time interval $[t_1, t_2]$ does not vary and equals λ_{t_1}, then the transition probability at times $0 \leq t_1 \leq t_2$ is given by

$$\mathsf{P}_{(X_{t_2}|X_{t_1})}(x_{t_2}|x_{t_1}) = \binom{x_{t_2}-1}{x_{t_1}-1} e^{-\lambda_{t_1}(t_2-t_1)x_{t_1}} (1-e^{-\lambda_{t_1}(t_2-t_1)})^{x_{t_2}-x_{t_1}} \quad \text{for } x_{t_2} = x_{t_1}, x_{t_1}+1, \ldots.$$

Let the stochastic process $\{Y_t,\, t \geq 0\}$ be the corresponding *partially-observable pure birth process*(PO-PBP), with the parameter vector (λ_t, p_t). Bean et al. [1] simplified Theorem Theorem 1 for a PO-PBP, as provided in Corollary 1.

Corollary 1 ([1]). *Consider a PO-PBP* $\{Y_t,\ t \geq 0\}$ *with the parameter vector* (λ_t, p_t), *and the underlying PBP* $\{X_t,\ t \geq 0\}$ *with the known initial population size of* x_0.

(i) The quantity ϱ_n^{ℓ} *for* $\ell = y_{t_n}, y_{t_n}+1, \ldots,$ *and* $n = 1, 2, \ldots,$ *is given by*

$$ey_n!\binom{\ell}{y_n} p_{t_n}^{y_n}(1-p_{t_n})^{\ell-y_n} \sum_{j=\overline{x}_{t_{n-1}}}^{\ell} \binom{\ell-1}{j-1} e^{-\lambda_{t_{n-1}}(t_n-t_{n-1})j}(1-e^{-\lambda_{t_{n-1}}(t_n-t_{n-1})})^{\ell-j} \varrho_{n-1}^{j},$$

where $\overline{x}_{t_n} := \max\{x_0, y_{t_1}, \ldots, y_{t_n}\}$. *The initial conditions are as provided in Theorem 1.*

(ii) The conditional p.m.f. of the next partial observation, given all past n *partial observations equals*

$$\mathsf{P}_{(Y_{t_{n+1}}|\boldsymbol{Y}_n)}(y_{t_{n+1}}|\boldsymbol{y}_n) = \frac{1}{\sum_{\ell=\overline{x}_{t_n}}^{\infty} \varrho_n^{\ell}} \left(\sum_{x_{t_{n+1}}=\overline{x}_{t_{n+1}}}^{\infty} \sum_{x_{t_n}=\overline{x}_{t_n}}^{x_{t_{n+1}}} \binom{x_{t_{n+1}}}{y_{t_{n+1}}} p_{t_{n+1}}^{y_{t_{n+1}}} (1-p_{t_{n+1}})^{x_{t_{n+1}}-y_{t_{n+1}}} \right.$$
$$\left. \times \binom{x_{t_{n+1}}-1}{x_{t_n}-1} e^{-\lambda_{t_n}(t_{n+1}-t_n)x_{t_n}} \left(1-e^{-\lambda_{t_n}(t_{n+1}-t_n)}\right)^{x_{t_{n+1}}-x_{t_n}} \varrho_n^{x_{t_n}} \right),$$

for $y_{t_{n+1}} = 0, 1, 2, \ldots,$ *and* $n = 1, 2, \ldots.$

An important question that may arise here is the dependency structure of the stochastic process $\{Y_t,\ t \geq 0\}$ which is addressed in $t \in (0, \infty)$. Theorem 2.

Theorem 2 ([1]). *The* `PO-CTMP` *process is not Markovian of any order. That is, for any fixed value of* $k = 1, 2, \ldots,$ *there exist* $0 \leq t_1 \leq \cdots \leq t_n$, $y_1, \ldots, y_n$, *and* $n > k$, *such that,*

$$\mathbf{Pr}(Y_{t_n} = y_{t_n} | Y_{t_1} = y_{t_1}, \cdots, Y_{t_{n-1}} = y_{t_{n-1}}) \neq \mathbf{Pr}(Y_{t_n} = y_{t_n} | Y_{t_{n-k}} = y_{t_{n-k}}, \cdots, Y_{t_{n-1}} = y_{t_{n-1}}).$$

Likelihood function. Although, Theorem 2 makes finding the likelihood function of a `PO-PBP` more challenging and complicated, one can use the chain rule along with Corollary 1 to construct the likelihood function:

$$\mathsf{L}_{\boldsymbol{Y}_n}(\boldsymbol{y}_n; \boldsymbol{\lambda}_n, \boldsymbol{p}_n) = \prod_{k=1}^{n} \mathsf{P}_{(Y_{t_k}|\boldsymbol{Y}_{k-1})}(y_{t_k}|\boldsymbol{y}_{k-1}), \tag{1}$$

where $\boldsymbol{\lambda_n} := (\lambda_0, \lambda_{t_1}, \ldots, \lambda_{t_n})$. Now, by having the likelihood function at hand, one can find the MLE of unknown parameters for a `PO-PBP`. However, there are some infinite sums involved with the likelihood function which should be handled carefully in numerical computations. One approach to deal with those infinite sums is to truncate them by exploiting Chebyshev's inequality. More precisely, Chebyshev's inequality prescribes to truncating the infinite sum over the realizations of the conditional random variable $(X_{t_n} \mid \boldsymbol{Y}_n = \boldsymbol{y}_n)$ at

$$\mathbb{E}\left[X_{t_n} \mid \boldsymbol{Y}_n = \boldsymbol{y}_n\right] + 20\sqrt{\mathsf{Var}(X_{t_n} \mid \boldsymbol{Y}_n = \boldsymbol{y}_n)}, \tag{2}$$

to guarantee that at least 99.75% of the corresponding probability distribution is covered. Bean et al. [2] derived those expected values involved in the truncation point (2) analytically.

Proposition 1 ([2]). *Consider a* PO-PBP $\{Y_t,\ t \geq 0\}$ *with the parameter vector* (λ_t, p_t), *and the underlying* PBP $\{X_t,\ t \geq 0\}$. *We have,*

$$\mathbb{E}\left[X_{t_n} \mid \boldsymbol{Y}_n = \boldsymbol{y}_n\right] = \frac{\overline{x}_{t_n} + (1 - p_{t_n})(1 - e^{-\lambda_{t_n} t_n})}{p_{t_n} + (1 - p_{t_n})e^{-\lambda_{t_n} t_n}},$$
$$\mathsf{Var}(X_{t_n} \mid \boldsymbol{Y}_n = \boldsymbol{y}_n) = \frac{(\overline{x}_{t_n} + 1)(1 - p_{t_n})(1 - e^{-\lambda_{t_n} t_n})}{(p_{t_n} + (1 - p_{t_n})e^{-\lambda_{t_n} t_n})^2},$$

where $\overline{x}_{t_n}$ *is as defined in Corollary 1.*

Prediction. In order to predict the future values of the process given the past partial observations, we use the MLE of the conditional expected value $\mathbb{E}\left[Y_{t_{n+1}} \mid \boldsymbol{Y}_n = \boldsymbol{y}_n\right]$. Due to the invariant property of MLEs, we only need to find the MLE of the unknown parameters λ_t and p_t and replace them in the equation provided in Proposition 2.

Proposition 2 ([2]). *Consider a* PO-PBP $\{Y_t,\ t \geq 0\}$ *with the parameter vector* (λ_t, p_t), *and the underlying* PBP $\{X_t,\ t \geq 0\}$. *We have,*

$$\mathbb{E}\left[Y_{t_{n+1}} \mid \boldsymbol{Y}_n = \boldsymbol{y}_n\right] = p_{t_{n+1}} e^{\lambda_{t_n}(t_{n+1} - t_n)} \mathbb{E}\left[X_{t_n} \mid \boldsymbol{Y}_n = \boldsymbol{y}_n\right],$$

where $\mathbb{E}\left[X_{t_n} \mid \boldsymbol{Y}_n = \boldsymbol{y}_n\right]$ *is as given in Proposition 1.*

References

[1] Bean NG, Elliott R, Eshragh A., Ross JV. On binomial observations of continuous-time Markovian population models. Journal of Applied Probability. 2015;52(2):457–472.

[2] Bean NG, Eshragh A, Ross JV. Fisher Information for a partially-observable simple birth process. Communications in Statistics-Theory and Methods. 2016;45(24):7161–7183.

[3] Elliott RJ, Aggoun L, Moore JB. Hidden Markov models: Estimation and control. New York: Springer; 1994.

[4] Renshaw E. Modelling biological populations in space and time. Cambridge: Cambridge University Press; 1993.
